# Physicochemical and Phytochemical Analyses of Copra and Oil of *Cocos nucifera* L. (*West Coast Tall* Variety)

**DOI:** 10.1155/2014/310852

**Published:** 2014-11-10

**Authors:** Probir Kumar Ghosh, Paramita Bhattacharjee, Souvik Mitra, Mousumi Poddar-Sarkar

**Affiliations:** ^1^Department of Food Technology and Biochemical Engineering (Centre for Advanced Study), Jadavpur University, Kolkata 700 032, India; ^2^Department of Botany (Centre for Advanced Study), University of Calcutta, 35-Ballygunge Circular Road, Kolkata 700 019, India

## Abstract

Coconut copra from *West coast tall* variety, cultivated in Kerala, India, was subjected to aqueous and solvent extractions (using *n*-hexane). Additionally, oil was extracted from the copra in Soxhlet assembly using petroleum ether (b.p. 60–80°C). Physicochemical and phytochemical analyses were conducted for the extracts and the oil, with commercial coconut oil as the experimental control. The physicochemical analyses showed that the aqueous extract of copra was milky-white in color with a sweet odor, while the solvent extract was pale yellow and odorless. The commercial oil had 0.08 ± 0.02% oleic acid and a TOTOX value of 7.73 ± 0.78, lower than the Soxhlet extracted oil. Among all the extracts and oils, best phytochemical properties, antioxidant activity (DPPH activity, IC_50_ value 0.04 ± 0.01 mg/mL), total phenol (0.96 ± 0.04 mg gallic acid eq./g dry copra), reducing power (40.49 ± 1.84 mg BHT eq./g dry copra), and anti-inflammatory activity (NO activity, IC_50_  value 0.77 ± 0.06 mg/mL) were obtained in the commercial coconut oil, followed by the Soxhlet extracted oil, aqueous extract, and solvent extract. Fatty acid composition analyses showed mainly medium chain fatty acids in the copra oil with lauric acid as the predominant fatty acid (51.88% and 44.84% in Soxhlet extracted and commercial oils, resp.).

## 1. Introduction 

Coconuts (*Cocos nucifera* Linn.) belonging to the family Arecaceae are cultivated mainly in the tropical areas of high humidity, regular rainfall, and sandy soil. Countries such as India, Sri Lanka, Indonesia, and Philippines have major share in the global production of coconuts. India is the largest producer of coconuts in the world (annual production of 16,943 million nuts in 2010-2011), and the* West coast tall* variety is one of the major varieties cultivated there [1, 2]. Copra is the dried coconut kernel with low moisture content (6–8%) and is used to obtain coconut oil by expellers and organic solvents [3]. Coconut oil is rich in medium chain fatty acids and exhibits good digestibility [4].

The sun-dried copra finds significant use in flavor and fragrance industries internationally as a source of primary flavoring from which the typical lactonic odor of coconuts is extracted [5]. Besides, studies on Nigerian variety of coconuts have established the copra to be a rich source of phytochemicals (such as phenols, flavonoids, glycosides, tannins, alkaloids, and saponins) which contribute to its antioxidant, anti-inflammatory and reducing power activities [6–8]. However, reports on characterization of the copra are scanty and to the best of our knowledge there is no report on the proximate, physicochemical, and phytochemical properties of the copra of* West coast tall* variety.

In our study, we have determined the proximate composition and the physicochemical and phytochemical properties of the* West coast tall* variety of copra. Proximate analysis of copra provides information on ash, moisture, crude fat, protein, crude fiber, and carbohydrate (by difference), while the physicochemical parameters mainly assess the color and odor. The phytochemical analyses include qualitative detection of alkaloids, flavonoids, glycosides, saponins, tannins, steroids, terpenoids, and acid compounds and quantitative determination of their therapeutic properties such as total phenol content, antioxidant, anti-inflammatory, and reducing power activities.

The present fat/oil based product development strategies are primarily focused on improvement of the fats and oils which constitute the products. These improvements are in three broad areas: application development, analytical development, and triglyceride replication [9]. For product development and design, especially for functional foods/nutraceuticals, it is necessary to analyze the physicochemical as well as the phytochemical properties of the constituent fats/oils.

In our work, the oil from copra has also been analyzed for both physicochemical and phytochemical properties. The physicochemical properties of coconut oil assayed included evaluation of parameters such as color, specific gravity, viscosity, acid value, peroxide value, and para-anisidine value. We have assessed the phytochemical properties of copra oil for the same parameters as mentioned above for copra. Gas chromatographic analyses of the coconut oil allow assessment of its fatty acid composition. Seneviratne and Dissanayake [10] have reported on fatty acid composition of coconut oil obtained from* ‘Ordinary tall'* variety of coconuts cultivated in Sri Lanka. However, to the best of our knowledge, no literature exists on fatty acid composition of oil extracted from* West coast tall* variety of coconut copra.

This work reports on proximate composition of coconut copra and physicochemical and phytochemical properties of the same and of the copra oil.

## 2. Materials and Methods

### 2.1. Materials

The coconut copra (ball copra) from* West coast tall* variety of coconuts were authenticated by Coconut Development Board, Kolkata, India, cultivated in the state of Kerala, India, and were purchased from a local supermarket of Kolkata, India. The agroclimatic requirements of coconut are coastal sandy soil with pH 5.5–7.0, mean temperature of cultivation 30 ± 5°C, and annual rainfall between 1000–3000 mm. The mature coconuts were harvested in the present year (2014), when they were 12 months old. The harvested coconuts were subsequently dried to obtain copra. All this information was collected from Coconut Development Board, Kolkata, India.

The copra were sorted and ground by an electric grinder (M/s Philips Electronics Ltd., Chennai, India) for extraction. Commercial coconut oil (M/s KPL Oil Mills (P) Ltd., Kerala, India) was purchased from a local supermarket of Kolkata (control oil sample). The commercial oil had been extracted from coconut copra of* West coast tall* variety cultivated and harvested under similar conditions and the oil was obtained using expeller method, confirmed by the manufacturer.

Specialty chemicals such as 1,1-diphenyl-2-picrylhydrazyl (DPPH), sodium nitroprusside, Griess reagent, gallic acid, and butylated hydroxytoluene (BHT) were procured from M/s Sigma-Aldrich Corp. (St. Louis, MO, USA). Methanol, toluene, *n*-hexane, petroleum ether (b.p. 60–80°C), sulphuric acid, Folin-Ciocalteu reagent, trichloroacetic acid, sodium carbonate, aluminum chloride, potassium ferricyanide, and sodium sulphate (anhydrous) were purchased from Merck (Darmstadt, Germany). All chemicals were of AR grade.

### 2.2. Proximate Analyses of Coconut Copra

Proximate analyses of coconut copra were carried out according to AOAC methods [11]. The analyses were conducted using standard protocols as follows: moisture content, ash, crude fat using Soxhlet assembly with petroleum ether (b.p. 60–80°C), crude protein by Kjeldahl method, crude fiber, and total carbohydrates by difference.

### 2.3. Aqueous and Solvent Extraction from Copra and Soxhlet Extraction of Coconut Copra Oil

The aqueous and solvent extracts from copra were obtained in accordance with the method reported by Odenigbo and Otisi [6], with few modifications. 5 g each of dried ground copra was soaked in distilled water and *n*-hexane (25 mL) for 24 h at 23 ± 2°C, for aqueous and solvent extractions, respectively.

Coconut oil was obtained using Soxhlet assembly with petroleum ether (b.p. 60–80°C) for 8 h. After extraction, the solvents were evaporated in a rotary vacuum evaporator at 45 mm bar vacuum (M/s Eyela Corp., Kyoto, Japan). The yield of extracts (aqueous and solvent) and Soxhlet extracted oil were obtained gravimetrically. All the samples were stored in amber colored screw capped glass vials in an inert atmosphere of nitrogen at −18°C in dark, until further analyses.

### 2.4. Analyses of Physicochemical Properties of Extracts of Copra and Oils

The study of physicochemical properties of extracts from copra included estimation of color and odor. For oils specific gravity, viscosity, and color were analyzed. The viscosity (Pa·s) of oils was measured using the Brookfield Digital DV-E Synchro-Electric Viscometer (M/s Brookfield Engineering Company, Middleborough, MA, USA) with spindle LV-1 at 23 ± 2°C in a speed range of 3–60 rpm. The color of oils was analyzed by Lovibond Tintometer Model-F, using 1 mL cuvette (M/s Tintometer Ltd., Salisbury, Wiltshire, UK) and the color intensity was expressed as *Y* + 5*R*.

AOAC [11] methods for study of chemical properties included assays of acid value (reported as free fatty acids (FFA), % oleic acid) and peroxide value (PV) (meq./kg oil). Para-anisidine value (p-AV) was analyzed by IUPAC [12] method and TOTOX value as 2PV + p-AV.

### 2.5. Qualitative and Quantitative Analyses of Extracts of Copra and Oils

For coconut copra extracts and oils, both qualitative and quantitative analyses of their phytochemicals were conducted. The qualitative detection of alkaloids, flavonoids, glucosides, saponins, resins, tannins, steroids, terpenoids, and acidic compounds was carried out according to the methods described by Odenigbo and Otisi [6]. The visual inspection of the intensity of color of precipitates (for extracts) and color of solution (for oils) was used to adjudge the levels of phytochemicals as high, medium, and low. The quantitative phytochemical analyses consisted of assays of antioxidant activity, total phenols, reducing power, and anti-inflammatory activities. These assays were conducted to analyze therapeutic properties of the extracts and the oils. The antioxidant activities of the extracts and the oils were determined by DPPH assay in accordance with Karakaya and Şimşek [13] and reported as IC_50_ values (mg/mL). Total phenol content was determined by the method described by Spanos and Wrolstad [14] using Folin-Ciocalteu reagent and reported as mg gallic acid eq./g dry copra, and reducing power was assayed according to the method described by Oyaizu [15] and expressed as mg BHT eq./g dry copra. The quantification of the phytochemicals was conducted from their respective standard curves. The anti-inflammatory activities were analyzed by* in vitro* nitric oxide (NO) scavenging activity and expressed as IC_50_ values (mg/mL) according to the method described by Correa et al. [16].

### 2.6. Fatty Acid Analysis of Oils

The fatty acid methyl ester (FAME) of oils was prepared according to the method described by Seneviratne and Dissanayake [10]. The gas chromatograph (GC) (CP 3800; M/s Varian Inc., Palo Alto, CA, USA) attached to WCOT, VF-1 MS Factor Four capillary column (15 m × 0.25 mm, i.d. 0.25 *μ*m) and equipped with flame ionization detector (FID) was used for the analyses. The injector and detector temperatures were 250°C and 260°C, respectively. The carrier gas was nitrogen at a flow rate of 1 mL/min. The oven temperature was programmed as follows: 70°C (1 min hold), 70°C to 230°C at 5°C/min, and final hold at 230°C (5 min). 1 *μ*L of FAME (dissolved in *n*-hexane) was injected into GC in splitless mode for analyses. Identification of fatty acids was performed using the standard 37-component FAME mix [butyric acid methyl ester (C_4:0_) − nervonic acid methyl ester (C_24:1n9_)] of M/s Supelco Analytical, St. Louis, MO, USA. The fatty acid composition was reported by the normalization method and expressed as % relative composition of individual fatty acids.

### 2.7. Statistical Analyses

Statistical analyses were carried out to analyze the effects of different extraction methods on the physicochemical and phytochemical properties. A *P* value of 0.05 was used to test the significance of all the tests. The statistical tests were conducted using STATISTICA 8.0 software (M/s Statsoft, OK, USA).

## 3. Results and Discussion

### 3.1. Proximate Composition Analyses of Copra

The proximate analyses of copra (on dry weight basis) revealed that it contained 3.94% moisture, 1.59% ash, 71.62% crude fat, 8.80% crude protein, 7.15% crude fiber, and 6.90% carbohydrates (by difference) (Table 1).

### 3.2. Yield and Characterization of Coconut Copra Extracts and Oil

The yield of *n*-hexane extract (0.327 g/g dry copra) of copra was higher than the aqueous extract (0.116 g/g dry copra), possibly due to higher solubility of the copra in *n*-hexane. The yield of coconut copra oil by Soxhlet assembly was 3.581 g/g dry copra.

### 3.3. Analyses of Physicochemical Properties of Extracts of Copra and Oils

The color of aqueous extracts was found to be milky-white, while the *n*-hexane extract was pale yellow. Although the aqueous extract had a sweet odor, the *n*-hexane extract was odorless. The Soxhlet extracted oil and commercial oil were both light yellow in color with typical characteristic coconut odors. The physicochemical properties of copra oil showed no significant differences between specific gravities of commercial oil (0.92 ± 0.01) and Soxhlet extracted oil (0.90 ± 0.01; *P* = 0.0700), % FFA (commercial oil: 0.08 ± 0.02, and Soxhlet extracted oil: 0.18 ± 0.02 oil; *P* = 0.3450), and Lovibond color indices (commercial oil: 5.17 ± 0.28, and Soxhlet extracted oil: 5.67 ± 0.29; *P* = 0.1010) (Table 2). These observations showed that the method of extraction of oil from coconut copra (either expeller pressed or Soxhlet) did not significantly affect the specific gravity, FFA, and color of the oils.

However, significant differences were obtained in viscosities of the oils at 23 ± 2°C (commercial oil: 0.04 ± 0.02 Pa·s, Soxhlet extracted oil: 0.03 ± 0.02 Pa·s;  *P* = 0.0110), peroxide values (commercial oil: 2.89 ± 0.30 meq./kg oil, Soxhlet extracted oil: 4.64 ± 0.44 meq./kg oil; *P* = 0.0049), p-anisidine values (commercial oil: 1.95 ± 0.31, Soxhlet extracted oil: 2.88 ± 0.34; *P* = 0.0252), and TOTOX values (commercial oil: 7.73 ± 0.78, Soxhlet extracted oil: 12.16 ± 1.23; *P* = 0.0062) (Table 2). Both the oils showed Newtonian flow, as is true for most edible oils. Higher peroxide and p-anisidine values were obtained in Soxhlet extracted oil than in the commercial oil, indicating higher primary and secondary oxidation of oil during Soxhlet extraction. Higher peroxide and p-anisidine values contributed to higher TOTOX values, which represents total oxidation of oil as was true for the Soxhlet extracted oil.

### 3.4. Qualitative Phytochemical Characterization of the Copra Extracts and Oils

From Table 3, we observe that the aqueous extract consisted of glycosides (in moderate intensity) and resins, saponins, and alkaloids (in low intensities). Flavonoids, tannins, steroids, terpenoids, and acidic compounds were absent in this extract, while the *n*-hexane extract contained glycosides (in moderate intensity), with low intensity of alkaloids, saponins, and resin (Table 4). These glycosides had been coextracted from copra during aqueous and solvent extractions and in the oils during Soxhlet extraction and expeller pressing. These were detected as red precipitates of moderate intensities using Fehling's solutions A and B. Our observations were in agreement with those of Odenigbo and Otisi [6], who also reported glycosides in moderate to high intensities in aqueous and *n*-hexane extracts of Nigerian coconuts. Tannins, steroids, flavonoids, terpenoids, and acidic compounds were absent in both the extracts. We observed that saponins and glycosides were present in moderate intensity and alkaloids and resins in low intensity in both the oils. However, flavonoids, tannins, steroids, terpenoids, and acidic compounds were absent in the oils (Table 5).

Overall, our observations showed that flavonoids, tannins, steroids, terpenoids, and acidic compounds were absent both in the extracts and in the oils. Our observations were in agreement with those obtained in the aqueous and *n*-hexane extracts of coconuts of Nigeria, wherein presence of alkaloids, glycosides, saponins, and resins has been reported by Odenigbo and Otisi [6]. These authors have also reported absence of flavonoids in both aqueous and solvent extracts, in agreement with our study. However, in contrast to the same report, we did not find tannins and terpenoids in the extracts. The absence of acidic compounds in either extract was in agreement with previous researchers [6, 17]. Since alkaloids, glycosides, and saponins were found to be present in medium-low ranges, we did not attempt to quantify the same.

### 3.5. Quantitative Phytochemical Characterization of the Copra Extracts and Oils

The phytochemical analyses of copra extracts and oils (Table 6) showed highest antioxidant activity in commercial oil (IC_50_  0.04 ± 0.01 mg/mL) followed by that in the Soxhlet extracted oil (IC_50_  0.09 ± 0.01 mg/mL), aqueous extract (IC_50_  1.96 ± 0.14 mg/mL), and *n*-hexane extract (IC_50_  49.98 ± 1.86 mg/mL). The highest quantity of total phenols was obtained in the commercial oil (0.96 ± 0.04 mg gallic acid eq./g dry copra), the Soxhlet extracted oil had 0.78 ± 0.05 mg gallic acid eq./g dry copra, and the lowest was found in the aqueous extract (0.08 ± 0.02 mg gallic acid eq./g dry copra). Total phenols were not detected in the *n*-hexane extract.

Highest reducing potential was found in commercial oil (40.49 ± 1.84 mg BHT eq./g dry copra), followed by that in Soxhlet extracted oil (23.11 ± 1.47 mg BHT eq./g dry copra), aqueous extract (4.05 ± 0.29 mg BHT eq./g dry copra), and *n*-hexane extract (1.51 ± 0.18 mg BHT eq./g dry copra). The anti-inflammatory activity was found to be best for commercial oil (IC_50_  0.77 ± 0.06 mg/mL) followed by Soxhlet extracted oil (IC_50_  2.17 ± 0.15 mg/mL), aqueous extract (IC_50_  107.85 ± 3.29 mg/mL), and *n*-hexane extract (IC_50_  434.52 ± 10.09 mg/mL). To the best of our knowledge, we did not find any report on quantification of phytochemicals for aqueous and *n*-hexane extracts of copra. In agreement with our findings on copra oil, previous researchers have also reported on phenolic acids and antioxidant and anti-inflammatory activities in coconut kernel oils [18, 19].

Our observations showed that the commercial copra oil (experimental control) showed best antioxidant, total phenol, anti-inflammatory, and reducing power activities, followed by the oil obtained by Soxhlet assembly, aqueous extract, and *n*-hexane extracts of copra. For oil samples, this trend is attributed to the differences in the methods of extraction of oil from copra. Since most phytochemicals are heat sensitive and expeller method of extraction is conducted at relatively lower temperature than Soxhlet extraction, higher phytochemical content has been observed in the expeller extracted oil than in Soxhlet extracted oil. A high correlation (*r* = 0.99) between antioxidant activity and reducing power for aqueous and *n*-hexane extracts, and for commercial and Soxhlet extracted oils, was obtained in our study. This correlation was in agreement with that reported by Račková et al. [20] who reported on correlation between antiradical and antioxidant activities of alkaloids isolated from* Mahonia aquifolium*.

### 3.6. Fatty Acid Analyses of Copra Oils by Gas Chromatography (GC)

**Figure 1:**
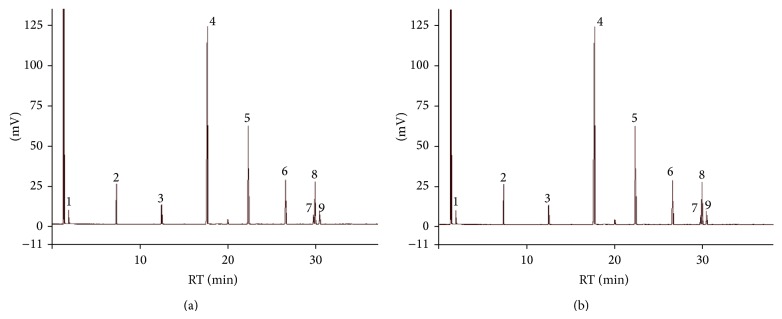
GC of FAME of (a) Soxhlet extracted oil from coconut copra, (b) commercial coconut oil. Peaks: (1) caproic acid (C_6:0_), (2) caprylic acid (C_8:0_), (3) capric acid (C_10:0_), (4) lauric acid (C_12:0_), (5) myristic acid (C_14:0_), (6) palmitic acid (C_16:0_), (7) linoleic acid (C_18:2_), (8) oleic acid (C_18:1_), (9) stearic acid (C_18:0_).

The GC chromatogram of FAME of copra oils shows the fatty acid composition of the Soxhlet extracted oil and the commercial oil (Figures 1(a) and 1(b), resp.).  Table 7 represents the retention times of the FAMEs on the GC column and the % relative composition of the fatty acids. FAME-GC analyzed the fatty acids of short chain (C_2_–C_6_), medium chain (C_8_–C_12_), and long chain (C_14_–C_18_) in coconut oil [9] against the Supelco standard of 37 FAMEs. This study showed that the most abundant fatty acid was lauric acid (C_12:0_) in the oil samples (44.84% in commercial oil and 51.88% in Soxhlet extracted oil). Therefore, medium chain fatty acids were found to be most predominant in the coconut oil samples which are reported to be health-beneficial. They are known to stimulate* Lactobacilli* [21] and possibly improve cognitive function in type I diabetes patients [22]. The percentage of lauric acid in the Soxhlet extracted copra oil was higher than that obtained from coconuts of Nigeria (41% reported by Odenigbo and Otisi [6]) and coconuts of Sri Lanka (49% reported by Seneviratne and Dissanayake [10]).

In our study, the ratio of saturated fatty acid (SFA) : (monounsaturated fatty acid) MUFA : polyunsaturated fatty acid (PUFA) in Soxhlet extracted oil was found to be 1 : 0.08 : 0.02 and 1 : 0.09 : 0.02 in the commercial oil, signifying these oils to be low in PUFA. Our results on fatty acid composition of copra oils were in agreement with Seneviratne and Dissanayake [10] who obtained similar results with coconut oil from coconut kernels cultivated in Sri Lanka. However, these oils do not meet the recommendation of The American Heart Organization which suggests 1 : 1 : 1 of SFA : MUFA : PUFA in edible fats and oils [23].

Since the copra oil contains mainly saturated fatty acids, the shelf stability of the same will be higher than the unsaturated oils. Also, owing to the same reason, flavor reversion (with storage) will be low as reported by O'Brien [9]. These findings suggest the oil obtained from copra to be a high stability oil (due to its oxidative and flavor stability). These high stability oils are desired in thermal food processing such as in frying and baking, and the copra oil studied would be suitable for these. The copra oil may also find use in triglyceride replication, in blending with other edible oils, and in formulating novel functional oils.

## 4. Conclusions

From our study we conclude that, among the copra extracts and oils, the commercial oil had the highest phytochemical content. A comparatively lower phytochemical content in the Soxhlet extracted oil indicated mechanical extraction to be more suitable method of oil extraction than solvent extraction. Since saturated fats are used in several food applications such as in confectionary and bakery products, we envisage potential applications of the expeller-pressed coconut copra oil in novel product development.

## Figures and Tables

**Table 1:** Proximate composition of coconut copra (*West coast tall* variety).

Analysis parameter	Composition (% on dry weight basis)
Moisture	3.94
Ash	1.59
Crude fat	71.62
Crude protein	8.80
Crude fiber	7.15
Carbohydrate (by difference)	6.90

**Table 2:** Physicochemical properties of the Soxhlet extracted coconut oil^e^.

Physicochemical property	Commercial oil	Soxhlet oil
Specific gravity	0.92 ± 0.01^a^	0.90 ± 0.01^a^
Lovibond color (Y + 5R)	5.17 ± 0.28^a^	5.67 ± 0.29^a^
Viscosity at 23 ± 2°C (Pa·s)	0.04 ± 0.02^c^	0.03 ± 0.01^b^
FFA (% oleic acid)	0.08 ± 0.02^b^	0.09 ± 0.02^b^
Peroxide value (PV) (meq./kg oil)	2.89 ± 0.30^c^	4.64 ± 0.44^d^
Para-anisidine value (p-AV)	1.95 ± 0.31^a^	2.88 ± 0.34^b^
TOTOX value (2 PV + p-AV)	7.73 ± 0.78^c^	12.16 ± 1.23^d^

^e^Results are mean ± SD of three independent experiments.

^a,b,c,d^Different letters in a row indicate significant difference at *P* < 0.05.

FFA, free fatty acids.

**Table 3:** Qualitative phytochemical analyses of coconut copra extract obtained by aqueous extraction.

Test	Observation	Inference	Intensity
(1) Alkaloids			
(a) Dragendorff reagent	Brick red precipitate	Alkaloids present	Low
(b) Wagner's reagent	Reddish brown precipitate	Alkaloids present	
(2) Flavonoids			
(a) Ammonium test	No yellow color change	Flavonoids absent	n.a.
(b) Aluminum chloride test	No yellow color change	Flavonoids absent	
(3) Glycosides	Dense red precipitate	Glycosides present	Moderate
(4) Saponins			
(a) Emulsion test	Emulsion formed	Saponins present	Moderate
(b) Frothing test	Stable froth formed	Saponins present	
(5) Resin			
(a) Precipitate test	White precipitate	Resins present	Moderate
(b) Color test	Light pink color	Resins present	
(6) Tannins			
(a) Ferric chloride test	No green precipitate	Tannins absent	n.a.
(7) Steroids			
(a) Conc. H_2_SO_4_ test	Reddish brown precipitate at interface	Steroids absent	n.a.
(8) Terpenoids			
(a) Conc. H_2_SO_4_ test	Red precipitate	Terpenoid absent	n.a.
(9) Acid compounds test			
(a) Moist litmus paper	No color change in moist blue litmus	Acid compounds absent	n.a.

n.a.: not applicable.

**Table 4:** Qualitative phytochemical analyses of coconut copra extract obtained by *n-*hexane.

Test	Observation	Inference	Intensity
(1) Alkaloids			
(a) Dragendorff reagent	Brick red precipitate	Alkaloids present	Low
(b) Wagner's reagent	Reddish brown precipitate	Alkaloids present	
(2) Flavonoids			
(a) Ammonium test	No yellow color	Flavonoids absent	n.a.
(b) Aluminum chloride test	No yellow color	Flavonoids absent	
(3) Glycosides	Dense red precipitate	Glycosides present	Moderate
(4) Saponins			
(a) Emulsion test	Emulsion formed	Saponins present	Low
(b) Frothing test	Stable froth formed	Saponins present	
(5) Resin			
(a) Precipitate test	White precipitate	Resins present	Low
(b) Color test	Light pink color	Resins present	
(6) Tannins			
(a) Ferric chloride test	No green precipitate	Tannins absent	n.a.
(7) Steroids			
(a) Conc. H_2_SO_4_ test	Reddish brown precipitate at interface	Steroids absent	n.a.
(8) Terpenoids			
(a) Conc. H_2_SO_4_ test	Red precipitate	Terpenoid absent	n.a.
(9) Acid compounds test			
(a) Moist litmus paper	No color change in moist blue litmus	Acid compounds absent	n.a.

n.a.: not applicable.

**Table 5:** Qualitative phytochemical analyses of coconut copra oil (commercial and by Soxhlet assembly).

Test	Observation	Inference	Intensity
(1) Alkaloids			
(a) Dragendorff reagent	Brick red color	Alkaloids present	Low
(b) Wagner's reagent	Reddish brown color	Alkaloids present	
(2) Flavonoids			
(a) Ammonium test	No yellow color	Flavonoids absent	n.a.
(b) Aluminum chloride test	No yellow color	Flavonoids absent	
(3) Glycosides	Dense red color	Glycosides present	Moderate
(4) Saponins			
(a) Emulsion test	Emulsion formed	Saponins present	Moderate
(b) Frothing test	Stable froth formed	Saponins present	
(5) Resin			
(a) Precipitate test	White precipitate	Resins present	Low
(b) Color test	Light pink color	Resins present	
(6) Tannins			
(a) Ferric chloride test	No green precipitate	Tannins absent	n.a.
(7) Steroids			
(a) Conc. H_2_SO_4_ test	Reddish brown precipitate at interface	Steroids absent	n.a.
(8) Terpenoids			
(a) Conc. H_2_SO_4_ test	Red precipitate	Terpenoid absent	n.a.
(9) Acid compounds test			
(a) Moist litmus paper	No color change in moist blue litmus	Acid compounds absent	n.a.

n.a.: not applicable.

**Table 6:** Quantitative phytochemical analyses of aqueous and solvent coconut copra extracts and oils^e^.

Sample type	Antioxidant activity (IC_50_ value of DPPH radical scavenging activity) (mg/mL)	Total phenol content (mg gallic acid eq./g dry copra)	Reducing power (mg BHT eq./g dry copra)	Anti-inflammatory activity (IC_50_ value of NO radical scavenging activity) (mg/mL)
Aqueous extract	1.96 ± 0.14^a^	0.08 ± 0.03^a^	4.05 ± 0.29^a^	107.85 ± 3.29^a^
*n-*Hexane extract	49.98 ± 1.86^b^	nd	1.51 ± 0.18^b^	434.52 ± 10.09^b^
Commercial oil	0.04 ± 0.01^c^	0.96 ± 0.04^b^	40.49 ± 1.84^c^	0.77 ± 0.06^c^
Soxhlet extracted oil	0.09 ± 0.01^d^	0.78 ± 0.05^c^	23.11 ± 1.47^d^	2.17 ± 0.15^d^

^e^Results are mean ± SD of three independent experiments.

nd: not detected.

^a,b,c,d^Different letters in a column indicate significant difference at *P* < 0.05.

**Table 7:** Fatty acid composition of coconut copra oils using gas chromatography.

Fatty acid	Retention time (min) (commercial oil)	Retention time (min) (Soxhlet extracted oil)	% relative composition	
			Commercial oil	Soxhlet extracted oil
C_6:0_ (caproic acid)	3.38	3.32	0.02	0.08
C_8:0_ (caprylic acid)	7.19	7.25	6.49	0.13
C_10:0_ (capric acid)	12.37	12.43	4.91	3.50
C_12:0_ (lauric acid)	17.52	17.55	44.84	51.88
C_14:0_ (myristic acid)	22.19	22.25	20.94	21.86
C_16:0_ (palmitic acid)	26.44	26.51	9.99	9.12
C_18:2_ (linoleic acid)	29.62	29.69	1.66	1.90
C_18:1_ (oleic acid)	29.78	29.85	7.64	8.82
C_18:0_ (stearic acid)	30.32	30.39	3.51	2.71
∑SFA			90.70	89.28
∑MUFA			7.64	8.82
∑PUFA			1.66	1.90

SFA, saturated fatty acids; MUFA, monounsaturated fatty acids; PUFA, polyunsaturated fatty acids.

## References

[1] Gopalakrishnan R. (2013). Global coconut scenario—India forges ahead. *Indian Coconut Journal*.

[2] Mannekote J. K., Kailas S. V. (2013). Value added products from coconut oil. *Indian Coconut Journal*.

[3] Canapi E. C., Augustin Y. T. V., Moro E. A., Pedrosa E., Bendano M. L. J., Shaihdi F. (2005). Coconut oil. *Edible Oil and Fat Products: Edible Oils, Bailey’s Industrial Oil and Fat Products*.

[4] Che Man Y. B., Marina A. M., Shaihdi F. (2006). Medium chain triglycerol. *Nutraceutical and Specialty Lipids and Their Co-Products*.

[5] Göhl B. (1981). Oil-bearing seeds and oil cakes. *Tropical Feeds: Feed Information Summaries and Nutritive Values*.

[6] Odenigbo U. M., Otisi C. A. O. (2011). Fatty acid and phytochemical contents of different coconut seed flesh in Nigeria. *International Journal of Plant Physiology and Biochemistry*.

[7] Wardlaw G. M. (2003). Lipids. *Contemporary Nutrition: Issues and Insights*.

[8] Whitney E., Rolfes S. R. (2008). The Lipids: Triglycerides, phospholipids and sterols. *Understanding Nutrition*.

[9] O'Brien R. D. (2003). Fats and oils formulation. *Fats and Oils: Formulating and Processing for Applications*.

[10] Seneviratne K. N., Dissanayake D. M. S. (2005). Effect of method of extraction on the quality of coconut oil. *Journal of Science—University of Kelaniya*.

[11] AOAC (2005). *Official Methods of Analysis*.

[12] IUPAC (1992). *Standard Methods for the Analysis of Oils, Fats and Derivates*.

[13] Karakaya S., Şimşek Ş. (2011). Changes in total polar compounds, peroxide value, total phenols and antioxidant activity of various oils used in deep fat frying. *Journal of the American Oil Chemists' Society*.

[14] Spanos G. A., Wrolstad R. E. (1990). Influence of processing and storage on the phenolic composition of Thompson seedless grape juice. *Journal of Agricultural and Food Chemistry*.

[15] Oyaizu M. (1986). Studies on product of browning reaction: antioxidative activities of products of browning reaction prepared from glucosamine. *Japan Journal of Nutrition*.

[16] Correa H., Valenzuela A. L., Ospina L. F., Duque C. (2009). Anti-inflammatory effects of the gorgonian *Pseudopterogorgia elisabethae* collected at the Islands of Providencia and San Andrés (SW Caribbean). *Journal of Inflammation*.

[17] Obidoa O., Joshua P. E., Eze N. J. (2010). Phytochemical analysis of *Cocos nucifera* L. *Journal of Pharmaceutical Research*.

[18] Intahphuak S., Khonsung P., Panthong A. (2010). Anti-inflammatory, analgesic, and antipyretic activities of virgin coconut oil. *Pharmaceutical Biology*.

[19] Marina A. M., Che Man Y. B., Nazimah S. A. H., Amin I. (2009). Antioxidant capacity and phenolic acids of virgin coconut oil. *International Journal of Food Sciences and Nutrition*.

[20] Račková L., Májeková M., Kostálová D., Štefek M. (2004). Antiradical and antioxidant activities of alkaloids isolated from *Mahonia aquifolium*. Structural aspects. *Bioorganic and Medicinal Chemistry*.

[21] Guerzoni M. E., Lanciotti R., Vannini L. (2001). Variability of the lipolytic activity in *Yarrowia lipolytica* and its dependence on environmental conditions. *International Journal of Food Microbiology*.

[22] Page K. A., Williamson A., Yu N. (2009). Medium-chain fatty acids improve cognitive function in intensively treated type 1 diabetic patients and support *in vitro* synaptic transmission during acute hypoglycemia. *Diabetes*.

[23] Gulla S., Waghray K. (2011). Effect of storage on physicochemical characteristic and fatty acid composition of selected oil blends. *Journal of Lipid Science*.

